# JAK inhibitors for refractory lymphoma

**DOI:** 10.18632/oncotarget.26054

**Published:** 2018-08-31

**Authors:** Hiroshi Kimura

**Affiliations:** Department of Virology, Nagoya University Graduate School of Medicine, Tsurumai-cho, Showa-Ku, Nagoya, Japan

**Keywords:** EBV, CAEBV, STAT3, ruxolitinib

Epstein-Barr virus (EBV), which belongs to the gammaherpesvirus subfamily, is a ubiquitous tumor virus. EBV infects B cells via CD21 and is associated with a variety of B-cell neoplasms such as Burkitt lymphoma, EBV-positive diffuse large B-cell lymphoma, and posttransplant lymphoproliferative disorders [1]. EBV also infects T and NK cells and is associated with extranodal NK/T cell lymphoma-nasal type, aggressive NK-cell leukaemia, and EBV-positive T-cell and NK-cell lymphoproliferative diseases of childhood (EBV+ T/NK LPD) [1, 2].

Chronic active EBV infection (CAEBV), which is defined as an EBV+ T/NK LPD in the 2017 WHO classification [3], is characterised by persistent EBV infection in T/NK cells and occasional transformation to peripheral lymphoma of T/NK-cell lineage cells [2, 4, 5]. The disease has an inflammatory aspect, as hypercytokinaemia is a common feature [6]. CAEBV is a rare but intractable disease with a poor prognosis. Compared with EBV+ B-cell neoplasms, which can be treated with rituximab-containing regimens, the optimum treatment strategy for CAEBV has not yet been established.

In this issue of *Oncotarget*, Onozawa *et al*. report that signal transducers and activators of transcription (STAT) 3 is constitutively active in both EBV+ T/NK cell lines and peripheral blood mononuclear cells from CAEBV patients [7]. They also show that ruxolitinib, an inhibitor of Janus kinase (JAK)1/2, suppresses not only the viability of EBV-infected T/NK cells but also their production of inflammatory cytokines. From these results, they conclude that STAT3 could be a therapeutic target for CAEBV.

STAT3 is an important signalling mediator and is constitutively activated in a variety of tumours, including lymphomas. Therefore, this mediator has been investigated as a target for the treatment of many types of cancer. Interestingly, STAT3 is activated in most EBV-associated hematological and epithelial malignancies. Latent membrane protein (LMP) 1 is an oncoprotein encoded by EBV, and constitutively activates the NF-κB, PI3K/AKT, and JNK pathways [1]. LMP1 specifically interacts with JAK and activates STAT proteins, including STAT3. Moreover, activation of the JAK/STAT pathway promotes the expression of LMP1 [8]. Onozawa *et al*. also demonstrate that ruxolitinib inhibits the phosphorylation of constitutively active STAT3 and induces apoptosis in an EBV-positive T-cell line. We recently reported that tofacinib, a JAK3-selective inhibitor, induces G1 cell-cycle arrest and inhibits the growth of EBV-positive T/NK lymphoma cells, in which the JAK/STAT pathway is activated [9]. Whether such constitutive activation of the JAK/STAT pathway is mediated by LMP1 is of great interest, although confirmation of this would require silencing of the *LMP1* gene, which is difficult, particularly in T/NK cell lines.

Novel approaches involving molecular-targeted therapies are needed to establish the optimum treatment strategy for CAEBV. At present the only curative therapy is hematopoietic stem cell transplantation, but this has a high incidence of complications [4, 5]. JAK inhibitors such as ruxolitinib and tofacinib are promising candidates for this purpose, as they target the EBV-driven pathway. More importantly, these drugs are approved and in use for other diseases (ruxolitinib for myelofibrosis and polycythemia vera; tofacinib for rheumatoid arthritis); therefore, evidence of their safety is abundant. We hope that incorporation of JAK inhibitors in the treatment regimen will improve the prognosis of CAEBV, although further extensive *ex vivo* and *in vivo* studies are necessary to clarify the efficacy of these drugs.
